# Validity of the relationship between mobility group box-1 protein and poor post–cardiac arrest neurological outcome during renal replacement therapy

**DOI:** 10.1186/s13054-018-2302-8

**Published:** 2019-03-12

**Authors:** Patrick M. Honore, David De Bels, Luc Kugener, Sebastien Redant, Rachid Attou, Andrea Gallerani, Herbert D. Spapen

**Affiliations:** 1 ICU Department, Centre Hospitalier Universitaire Brugmann-Brugmann University Hospital, Place Arthur Van Gehucthen, 4 1020 Brussels, Belgium; 2 Ageing & Pathology Research Group, Vrije Universiteit Brussel, 101 Laarbeeklaan, 1070 Brussels, Belgium

Recently in *Critical Care*, Sugita et al. confirmed the previously reported positive association between high HMGB-1 (high-mobility group box-1) levels and a poor neurological outcome in post–cardiac arrest (post-CA) patients [1]. Although we applaud this excellent study, we would like to make some comments. Brain damage after out-of-hospital CA is a leading cause of mortality and long-term neurological disability in survivors. Primary injury is caused by an immediate shutdown of cerebral blood flow. In the hours and days following CA, systemic ischemia/reperfusion (I/R)-induced processes may culminate in secondary neuronal cell death [2]. HMGB-1, a typical damage-associated molecular pattern protein, is crucial in inflammation and is critically involved in thrombosis and also mediates systemic organ I/R. Interestingly, almost half of the patients resuscitated after CA may develop acute kidney injury (AKI). More than 75% of AKI episodes occurred within 3 days after CA and about one third of these patients eventually needed continuous renal replacement therapy (CRRT) [3]. Sugita et al. did not mention the incidence of AKI or need of (C)RRT. However, given the high number of patients in shock (75% of patients with a Glasgow–Pittsburgh Cerebral Performance Categories Scale (CPC) score of 3 to 5 were on vasopressors!), it is reasonable to assume that a substantial number developed AKI or needed (C)RRT or both. With a molecular weight of 30 kDa, HMGB-1 is not eliminated by convective CRRT. However, Yumoto et al. showed that more than 90% of HMGB-1 was cleared from the circulation by highly adsorbent surface-treated polyacrylonitrile and polymethylmethacrylate membranes but that no such removal was observed with the less adsorptive polysulfone membranes [4]. Although polysulfone hemofilters are most commonly used for CRRT in Japan [5], implementing highly adsorbent filters would result in much lower serum HMGB-1 levels in post-CA patients. This not only would challenge the conclusions made by Sugita et al. but also could affect patient outcome since an extensive elimination of HMGB-1 may theoretically subdue the I/R-related post-CA injurious process.

## Abbreviations

AKI: Acute kidney injury
CA: Cardiac arrest
(C)RRT: (Continuous) renal replacement therapy
HMGB-1: High-mobility group box-1
I/R: Ischemia/Reperfusion
