# Supplementary material for: Validity of the relationship between mobility group box-1 protein and poor post–cardiac arrest neurological outcome during renal replacement therapy
Source: Crit Care. 2019 Mar 12;23:84. doi: 10.1186/s13054-018-2302-8 (PMC6419494; doi:10.1186/s13054-018-2302-8)
Supplement: Related texts

# Reply: Authors’ response

**Authors:** Atsushi Sakurai, Kosaku Kinoshita, Atsunori Sugita, Nobutaka Chiba, Junko Yamaguchi, Tsukasa Kuwana, Nami Sawada, Satoshi Hori

Following the revision of our data, we identified 12 of 128 patients to be treated with continuous renal replacement therapy (CRRT), including 7 who had been on haemodialysis therapy prior to the cardiac arrest (CA). Only 5 patients (3.9 %) were treated with CRRT for renal failure in our study.

Whether elimination of HMGB1 could contribute to the amelioration of the neurological deficits in patients with post-cardiac arrest syndrome (PCAS) is a crucial question. HMGB1 clearance from the brain may afford brain protection in PCAS patients. Considering the small molecular weight of HMGB1 and that CA induces the blood–brain barrier breakdown in the early phase of post-resuscitation, it is possible that serum HMGB1 may invade the brain through the broken BBB and promote the inflammatory pathophysiology of the ischemic reperfusion brain injury. Xu et al. reported that the neurological deficits significantly improved in rats resuscitated from CA following the injection of the anti-HMGB1 antibody into their cerebral ventricles [1]. Another study reported that the intravenous injection of an anti-HMGB1 antibody improved the cerebral haemorrhage-induced neurological deficits in rats [2]. Therefore, these findings suggest that the systemic clearance of HMGB1 might also ameliorate post-cardiac arrest brain injury.

As Dr. Honore proposed, using the CRRT for clearance of serum HMGB1 may be a promising candidate method for the treatment of PCAS patients. During the process of brain injury, damage-associated molecular patterns, including HMGB1, are released from the injured neurons and activate inflammation, which results in further neural and glial death during stroke [3]. In the present study, the peaks of serum HMGB1, interleukin-6 and neuron-specific enolase were 0, 24 and 48 h from resuscitation, respectively [4], indicating that a pathologic mechanism similar to stroke may occur in PCAS patients. In contrast HMGB1 was higher in the cerebrospinal fluid of PCAS patients than in their serum at 48 h after resuscitation [5]. These results suggest that, in addition to the primary inflammatory brain injury, HMGB1 may also promote the secondary inflammatory brain injury in PCAS patients. However, when the primary brain damage is severe, decreasing HMGB1 activity in PCAS patients will not be effective. Therefore, both the timing of the treatment and the severity of brain injury should be taken into consideration when investigating HMGB1 clearance treatment therapy for PCAS patients.
